# 含维奈克拉、阿糖胞苷和高三尖杉酯碱减瘤预处理allo-HSCT治疗伴RUNX1::RUNX1T1融合基因的复发难治急性髓系白血病疗效及安全性分析

**DOI:** 10.3760/cma.j.cn121090-20250312-00133

**Published:** 2026-02

**Authors:** 子璐 张, 承伟 金, 肃 李, 璐祥 王, 佳瑜 黄, 传和 江, 海洋 陆, 晓霞 胡

**Affiliations:** 1 上海交通大学医学院附属瑞金医院血液科，上海血液学研究所，国家转化医学中心，上海 200025 National Research Center for Translational Medicine, Shanghai Institute of Hematology, Ruijin Hospital, Shanghai JiaoTong University School of Medicine, Shanghai 200025, China; 2 上海力泉医院，上海 201418 Shanghai Liquan Hospital, Shanghai 201418, China

## Abstract

回顾性分析2023年3月至2024年12月在上海交通大学医学院附属瑞金医院血液科接受含维奈克拉、阿糖胞苷和高三尖杉酯碱减瘤预处理桥接异基因造血干细胞移植（allo-HSCT）的5例伴RUNX1::RUNX1T1融合基因复发难治急性髓系白血病（AML）患者的临床结局。5例患者诊断至allo-HSCT中位时间为315（217～560）d，移植后6个月所有患者RUNX1::RUNX1T1融合基因定量转阴，中位转阴时间移植后2（1～6）个月。中位随访时间625（372～1 010）d，所有患者无病生存，无流式细胞术或分子学可测量残留病阳性事件，初步提示含维奈克拉、阿糖胞苷和高三尖杉酯碱减瘤预处理作为伴RUNX1::RUNX1T1融合基因复发难治AML移植前桥接方案安全有效。

## Abstract (en)

Clinical data from five patients with refractory/relapsed acute myeloid leukemia（AML）and RUNX1::RUNX1T1 fusion gene, treated with venetoclax, cytarabine, and homoharringtonine-based cytoreductive therapy before allogeneic hematopoietic stem cell transplantation（allo-HSCT）at Ruijin Hospital between March 2023 and December 2024, were retrospectively reviewed. The median time from diagnosis to allo-HSCT was 315 days（range: 217–560）. All patients achieved full donor chimerism by day 30 post-allo-HSCT. Within 6 months after transplant, the RUNX1::RUNX1T1 fusion gene was undetectable in all patients, with a median time to negative conversion of 2 months（range: 1–6 months）. The median follow-up time was 625 days（range: 372–1 010）. All patients remained disease-free, with no events of measurable residual disease（MRD）positivity by flow cytometry or molecular analysis documented. These findings preliminarily confirm that venetoclax, cytarabine, and homoharringtonine-based cytoreductive therapy is a safe and effective bridging therapy for allo-HSCT in patients with refractory/relapsed AML and RUNX1::RUNX1T1 fusion gene.

伴RUNX1::RUNX1T1融合基因急性髓系白血病（AML）占初发白血病的5％～10％[1]，对中大剂量阿糖胞苷治疗敏感，诱导缓解率高，分别被欧洲白血病网（ELN）2017和2022分类归入预后良好组[2]–[3]。但该亚型异质性强，强化疗复发率30％～50％，复发后再缓解率33％[4]，中位生存期仅9（3.68～14.32）个月[5]。我国异基因造血干细胞移植（allo-HSCT）专家共识推荐第二次巩固治疗后RUNX1::RUNX1T1融合基因定量下降<3 log的患者接受allo-HSCT[6]。但移植前RUNX1::RUNX1T1融合基因定量下降<1 log的患者移植后复发率显著增高[7]。如何进一步优化移植前RUNX1::RUNX1T1融合基因阳性AML患者的预处理方案是亟待解决的临床难题。基于维奈克拉的方案是复发难治AML移植前有效的桥接方案，在维奈克拉基础上联合高三尖杉酯碱（HHT）能显著提高伴RUNX1::RUNX1T1融合基因的复发难治AML再缓解率[8]。进一步研究发现，维奈克拉联合HHT作为复发难治AML的移植前桥接方案，有效降低移植前白血病负荷，提高移植后生存率[9]。本研究对我中心应用含维奈克拉、阿糖胞苷和HHT减瘤方案预处理allo-HSCT的5例伴RUNX1::RUNX1T1融合基因复发难治AML患者进行回顾性分析，探讨其临床安全性和疗效。

## 病例与方法

1. 病例：回顾性分析2023年3月至2024年12月上海交通大学医学院附属瑞金医院血液科采用含维奈克拉、阿糖胞苷和HHT方案桥接allo-HSCT的5例伴RUNX1::RUNX1T1融合基因复发难治AML患者的临床资料，结合骨髓细胞形态学、白血病免疫分型、细胞遗传学及分子生物学，依据2022年第五版《WHO造血和淋巴组织肿瘤分型标准》[10]和2023年中国复发难治性AML诊疗指南[11]诊断。5例患者均经“7+3”方案诱导缓解，后以中剂量阿糖胞苷巩固治疗，完成巩固治疗中位4（3～5）个疗程。3例患者巩固治疗中复发，2例流式细胞术检测的可测量残留病（MRD）由阴性转为阳性。

2. 预处理方案：维奈克拉100 mg −15 d，200 mg −14 d，400 mg −13 d～−7 d；阿糖胞苷100 mg/m^2^，−13 d～−7 d；HHT 1 mg/m^2^，−13 d～−7 d；氟达拉滨30 mg/m^2^，−6 d～−2 d；马法兰50 mg/m^2^，−6 d～−5 d；白消安3.2 mg/kg，−4 d～−3 d。

3. 移植物抗宿主病（GVHD）预防：急性GVHD（aGVHD）预防以环孢素+霉酚酸酯+短程甲氨蝶呤为主，亲缘单倍体（HID）和无关全相合（MUD）移植加用7.5～10 mg/kg抗人胸腺细胞球蛋白。

4. 疗效和不良反应：移植后1、2、3、6、9、12、18和24个月分别进行疗效评估，包括骨髓细胞形态学、流式细胞术MRD和RUNX1::RUNX1T1定量检测。疗效评估依据2022ELN标准[2]，RUNX1::RUNX1T1定量检测采用qPCR法，内参基因为ABL，检测灵敏度为10^−5^。

不良事件参考美国CTCAE第五版，主要评估血液学及非血液学不良反应。

5. 随访：采用门诊就诊方式进行随访，随访截止时间为2025年12月13日。

6. 统计学处理：计数资料以例数表示，计量资料以中位数（范围）表示。

## 结果

1. 临床特征：5例患者中位年龄35（26～60）岁。诊断时骨髓原始细胞比例中位数为33.0％（27.3％～68.5％），中位RUNX1::RUNX1T1融合基因定量449％（314％～623％）。患者临床资料详见表1。3例患者合并多种基因突变，其中1例合并c-KIT（D820位点）突变。诊断至allo-HSCT中位时间为315（217～560）d。3例复发患者RUNX1::RUNX1T1定量明显升高，4例移植前出现新分子生物学改变。

**表1:** 5例伴RUNX1::RUNX1T1融合基因的复发难治急性髓系白血病患者移植前临床资料

例号	初诊基因突变	初诊细胞遗传学	C2评估	移植前评估	移植前基因突变
1	RUNX1::RUNX1T1（345％）, KIT-TKD, ASXL3, ASXL2	46, XY	形态学CR， 流式MRD 0.005％ RUNX1::RUNX1T1/ABL 0.153％	形态学CR， 流式MRD 0.02％ RUNX1::RUNX1T1/ABL 1.15％	RUNX1::RUNX1T1, KIT-TKD
2	RUNX1::RUNX1T1（314％）, ASXL1, TET2	45, X, −X, t（8;21）（q22;q22）	形态学CR， 流式MRD 0.02％ RUNX1::RUNX1T1/ABL 0.56％	原始细胞41.5％， 流式MRD 26.62％ RUNX1::RUNX1T1/ABL 266％	RUNX1::RUNX1T1, KIT-TKD, ASXL1, TET2
3	RUNX1::RUNX1T1（623％）	44-46, X, del（Xq24）,add（8p23）, 21ps+	形态学CR， 流式MRD阴性 RUNX1::RUNX1T1/ABL 0.013％	原始细胞31％， 流式MRD 9.39％ RUNX1::RUNX1T1/ABL 257％	RUNX1::RUNX1T1, SF3B1, RAD21, ASXL2, WT1
4	RUNX1::RUNX1T1（465％）, FLT3-TKD, SMC3, ETV6, BCORL1, KDM6A, SMC1A	43-45, X, −X, t（8;21）（q22;q22）	形态学CR， 流式MRD阴性 RUNX1::RUNX1T1/ABL 0.06％	形态学CR， 流式MRD 0.01％ RUNX1::RUNX1T1/ABL 0.25％	RUNX1::RUNX1T1, DNMT3A, BCORL1
5	RUNX1::RUNX1T1（449％），CSF3R	44-45, X, −Y, t（8;21）（q22;q22）	形态学CR， 流式MRD阴性 RUNX1::RUNX1T1/ABL 0.638％	原始细胞51％， 流式MRD 28.44％ RUNX1::RUNX1T1/ABL 366％	RUNX1::RUNX1T1, CSF3R, DHX15

**注** C2：第二次巩固治疗后；CR：完全缓解；流式MRD：流式细胞术检测的可测量残留病

2. 疗效分析：4例患者接受HID-HSCT，1例接受MUD-HSCT。CD34^+^细胞中位输注量为5.9（4.2～9.0）×10^6^/kg；中位中性粒细胞和血小板植入时间分别为12（11～15）d和14（10～25）d，移植后30 d患者均完全供者嵌合。1例合并c-KIT突变患者于移植后2个月启动阿伐替尼维持治疗（100 mg/d）。5例患者RUNX1::RUNX1T1融合基因均在移植后6个月内转阴，中位转阴时间2（1～6）个月。中位随访时间625（372～1 010）d，所有患者均无病生存，无患者出现流式细胞术或分子学MRD转阳。2例患者合并Ⅱ～Ⅳ度aGVHD，2例患者合并轻中度慢性GVHD，均经一线治疗后好转（表2）。

**表2:** 5例伴RUNX1::RUNX1T1融合基因的复发难治急性髓系白血病患者移植临床资料

例号	移植类型	CD34^+^细胞（×10^6^/kg）	单个核细胞（×10^8^/kg）	粒细胞植入时间（d）	血小板植入时间（d）	嵌合状态	RUNX1::RUNX1T1/ABL转阴时间	随访时间（d）	GVHD
1	HID	9.0	7.51	14	25	完全供者嵌合	移植后2个月	372	无
2	MUD	4.2	11.26	11	14	完全供者嵌合	移植后6个月	625	无
3	HID	5.5	8.40	15	17	完全供者嵌合	移植后1个月	821	Ⅳ度aGVHD中度cGVHD
4	HID	5.9	10.84	11	10	完全供者嵌合	移植后1个月	585	Ⅱ度aGVHD
5	HID	6.6	5.70	12	13	完全供者嵌合	移植后2个月	1010	轻度cGVHD

**注** aGVHD：急性移植物抗宿主病；cGVHD：慢性移植物抗宿主病；HID：亲缘单倍体供者；MUD：HLA全相合非血缘供者

3. 安全性分析：5例患者均按计划完成预处理，2例患者出现4级胃肠道不良反应。移植过程中粒细胞缺乏时间18（15～20）d，其中2例发生血流感染（分别检出大肠埃希菌、屎肠球菌），抗感染治疗后好转。1例患者出现心肌酶一过性升高，2例患者合并4级口腔黏膜炎。

## 讨论

尽管AML伴RUNX1::RUNX1T1属于预后良好AML，但临床异质性强，二级分子生物学突变、附加细胞遗传学异常及临床特征等与疾病的异质性密切相关[12]–[13]。基于蒽环类药物联合阿糖胞苷的治疗方案使87％～89％患者获得完全缓解[14]，氟达拉滨、维奈克拉、HHT、去甲基化药物、酪氨酸激酶抑制剂、CD33单抗等一定程度提高缓解率[15]–[16]，但不能降低复发率。复发是伴RUNX1::RUNX1T1 AML治疗失败的主要原因，长期随访结果显示复发率在30％～50％，通常在结束化疗1年内发生，从分子学复发至形态学复发中位时间仅0.8个月[17]。因此，allo-HSCT是2个疗程巩固强化后流式细胞术MRD阳性或基因下降不足3 log患者最佳巩固治疗，但仍有20％患者移植后复发[18]–[19]，复发后1年无复发生存率仅53％[20]。

缓解深度与移植后复发显著相关[17]。欧洲血液和骨髓移植学会分析第二次完全缓解后接受allo-HSCT的RUNX1::RUNX1T1融合基因AML患者，流式MRD阳性移植后复发率更高[21]。一项纳入631例接受allo-HSCT的t（8;21）AML回顾性分析进一步证实，移植前RUNX1::RUNX1T1阳性患者累积复发率显著高于RUNX1::RUNX1T1阴性患者（25.8％对13.5％，*P*<0.001）[22]。因此，如何提高伴RUNX1::RUNX1T1融合基因AML移植前缓解深度仍是临床亟待解决的关键问题。

在伴RUNX1::RUNX1T1融合基因AML患者中，维奈克拉为基础的治疗方案疗效欠佳。HHT是蛋白质合成抑制剂，通过特异性抑制NF-κB-MYC信号通路下调KIT表达，在伴RUNX1::RUNX1T1融合基因AML中具有良好的抗肿瘤活性[23]。HHT与蒽环类/阿糖胞苷联用提高伴t（8;21）AML诱导缓解率[24]，与维奈克拉联用通过抑制MCL-1及BCL-2协同促进白血病细胞凋亡[25]。刘启发教授团队发现HHT联合维奈克拉及阿扎胞苷可协同抑制MCL1、BCL-XL及BAX，在复发难治AML中总缓解率达79.1％（68.6％～85.4％）[9]。一项回顾性队列分析表明，维奈克拉联合阿扎胞苷基础上加用HHT可显著提高伴t（8;21）AML缓解率[26]。多中心队列研究证明，在维奈克拉、阿扎胞苷基础上联合HHT可显著提高伴RUNX1::RUNX1T1融合基因AML缓解率（69.2％对10％, *P*＝0.013）[27]。

基于联合用药分子生物学基础及临床疗效，本中心采用维奈克拉、阿糖胞苷联合HHT作为移植前预处理方案，5例患者均按计划完成治疗，中性粒细胞、血小板植入时间与非桥接治疗历史队列相当[28]。粒细胞缺乏时间虽有延长，但并未增加严重不良事件发生。在移植后评估中，该方案显著提高移植后RUNX1::RUNX1T1基因转阴率。截至末次随访，5例患者均实现长期分子学缓解。本研究样本量少，是一项探索性研究，随访时间较短，基于维奈克拉、阿糖胞苷和HHT的桥接方案是否能适用于其他复发难治AML，还有待前瞻性临床研究验证。
